# Supplementary material for: Impact of Chromosomal Context on Origin Selection and the Replication Program
Source: Genes (Basel). 2022 Jul 14;13(7):1244. doi: 10.3390/genes13071244 (PMC9318681; doi:10.3390/genes13071244)
Supplement: Supplementary file 1 [file genes-13-01244-s001.zip › genes-1702322-supplementary.pdf]

Figure S1

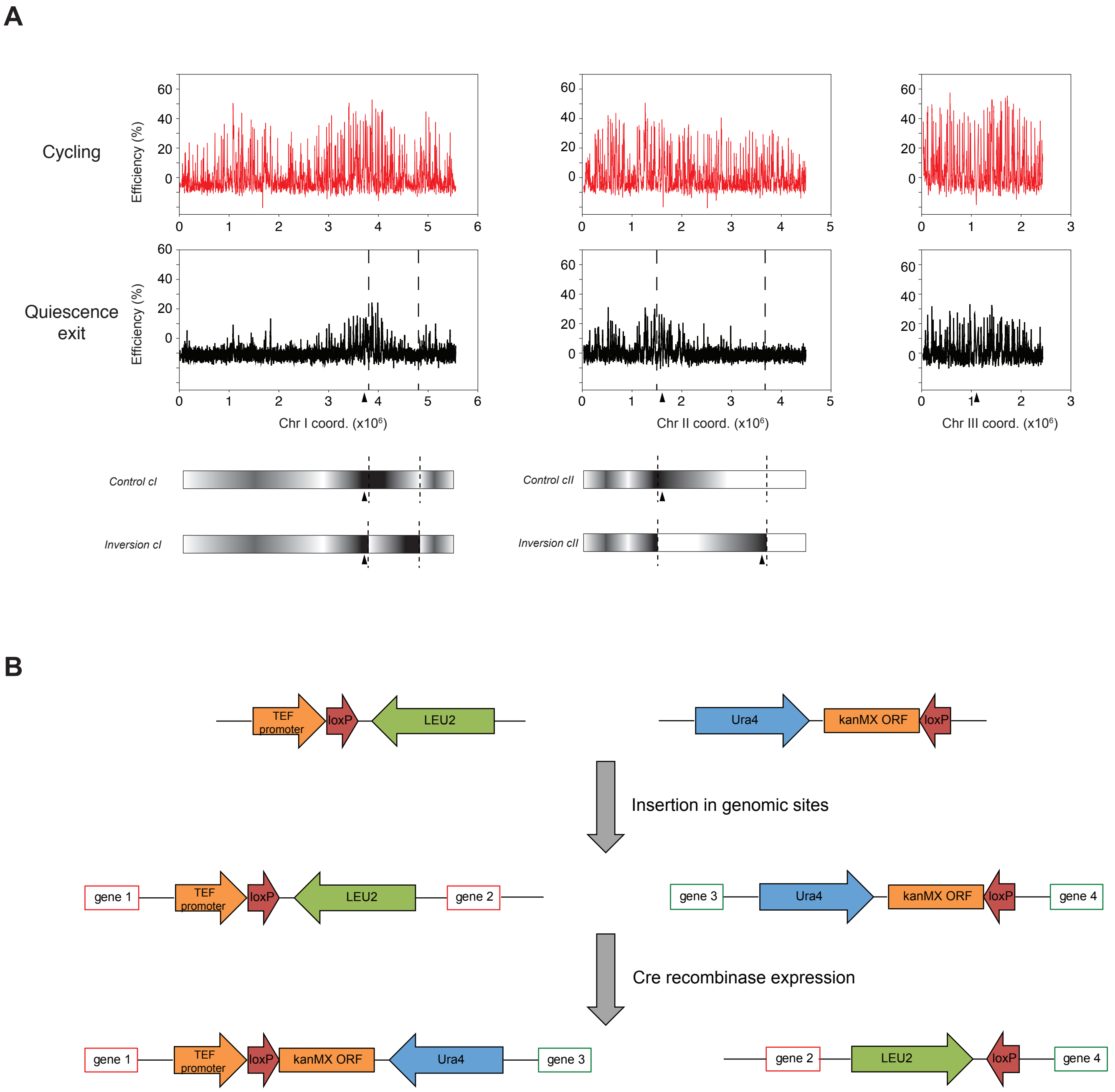

A
Cycling
Quiescence exit
Efficiency (%)
Chr I coord. (x10⁶)
Chr II coord. (x10⁶)
Chr III coord. (x10⁶)
Control cI
Inversion cI
Control cII
Inversion cII
B
TEF promoter
loxP
LEU2
Ura4
kanMX ORF
Insertion in genomic sites
gene 1
gene 2
gene 3
gene 4
Cre recombinase expression

Figure S1

C

ChrI: 3810766

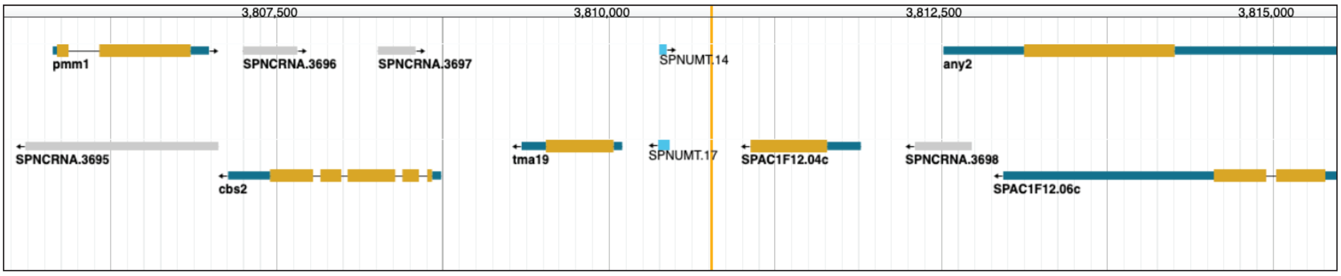

ChrI: 4785016

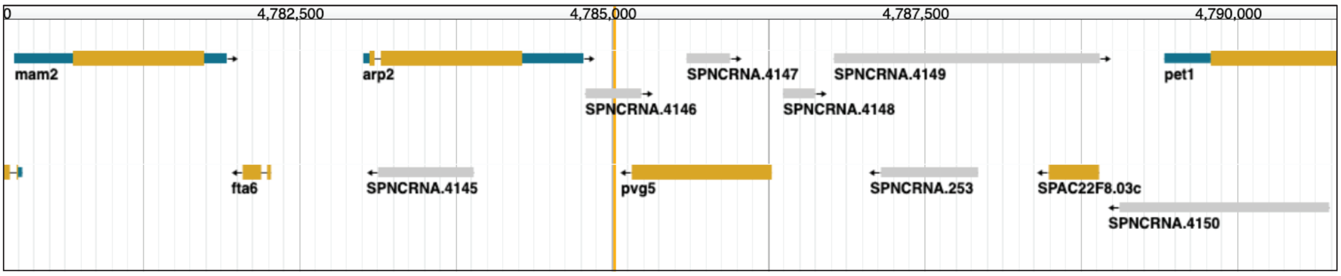

ChrII: 1577150

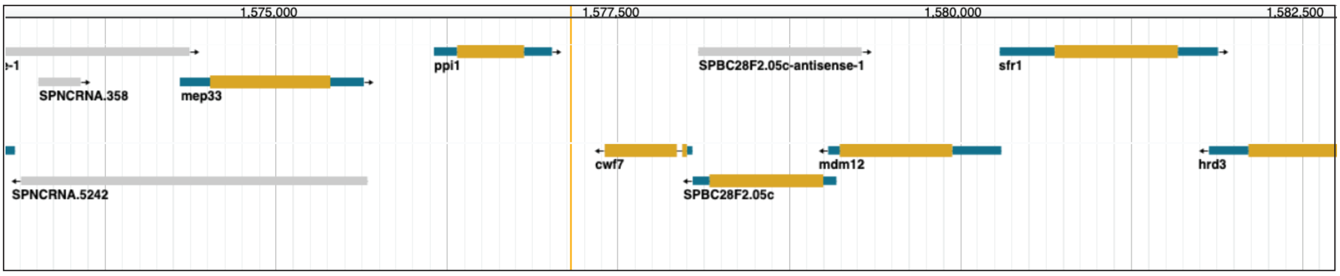

ChrII: 3694900

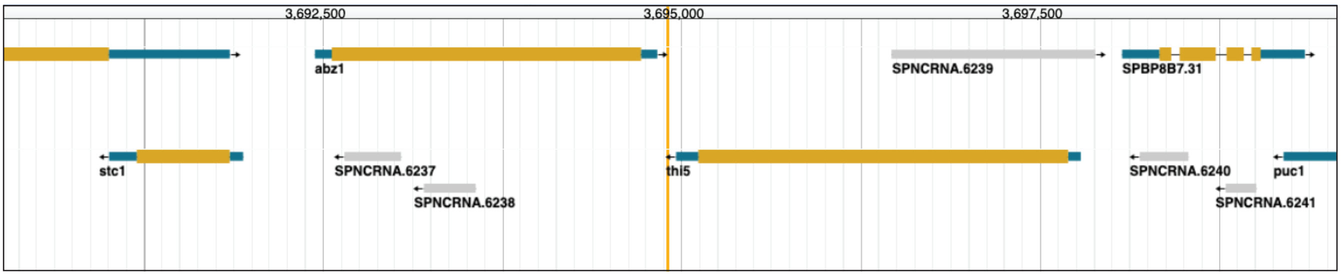

Figure S2

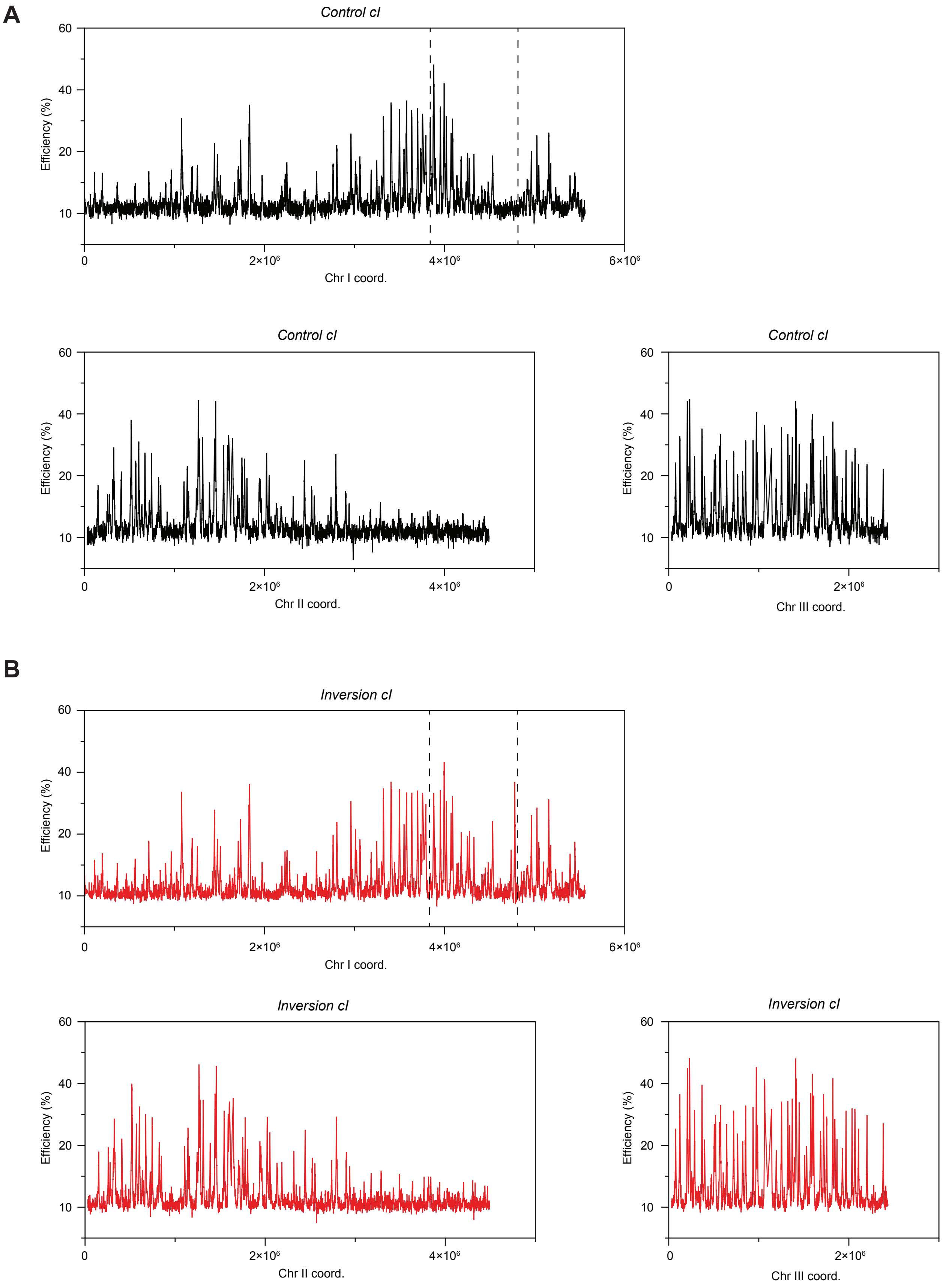

A
Control cI
Efficiency (%)
60
40
20
10
0
2×10⁶
4×10⁶
6×10⁶
Chr I coord.
Control cI
Chr II coord.
Control cI
Chr III coord.
B
Inversion cI
Chr I coord.
Inversion cI
Chr II coord.
Inversion cI
Chr III coord.

Figure S2

C

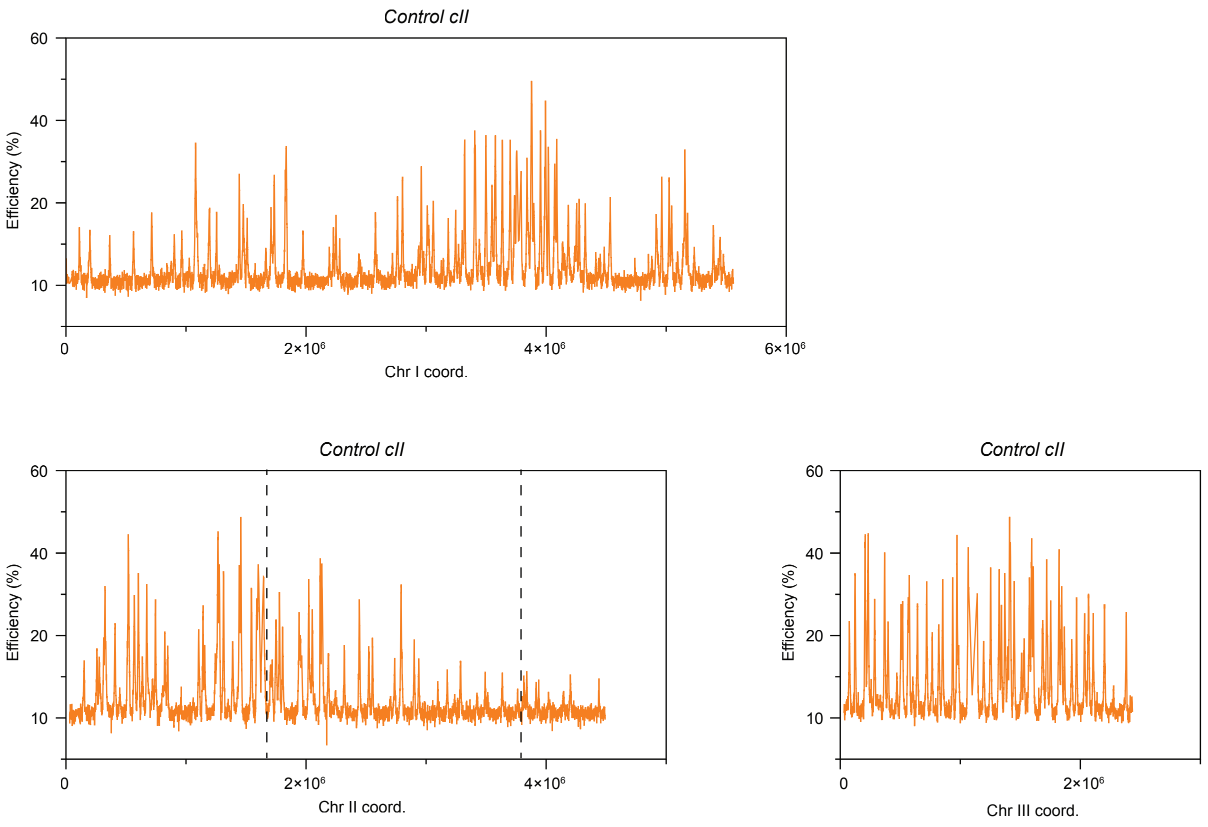
Control cII
Efficiency (%)
60
40
20
10
0
2×10⁶
4×10⁶
6×10⁶
Chr I coord.
Chr II coord.
Chr III coord.

D

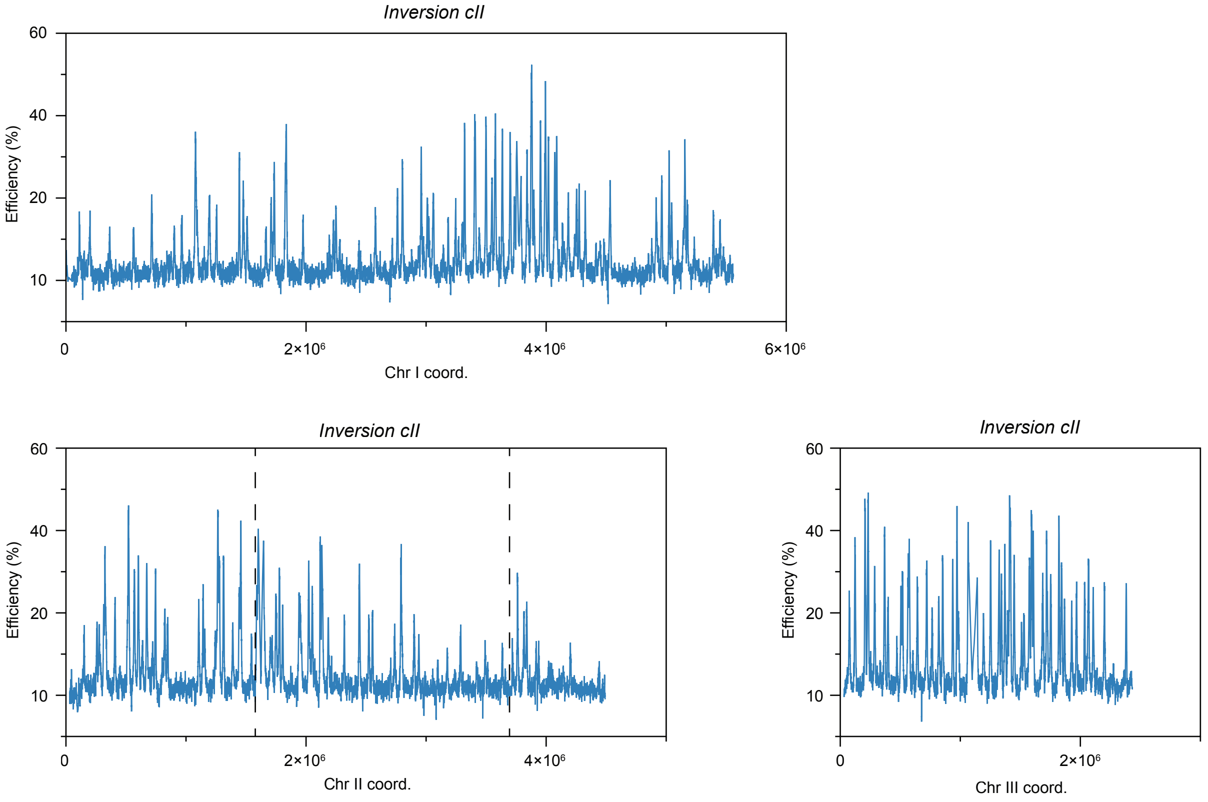
Inversion cII
Efficiency (%)
60
40
20
10
0
2×10⁶
4×10⁶
6×10⁶
Chr I coord.
Chr II coord.
Chr III coord.

Figure S3

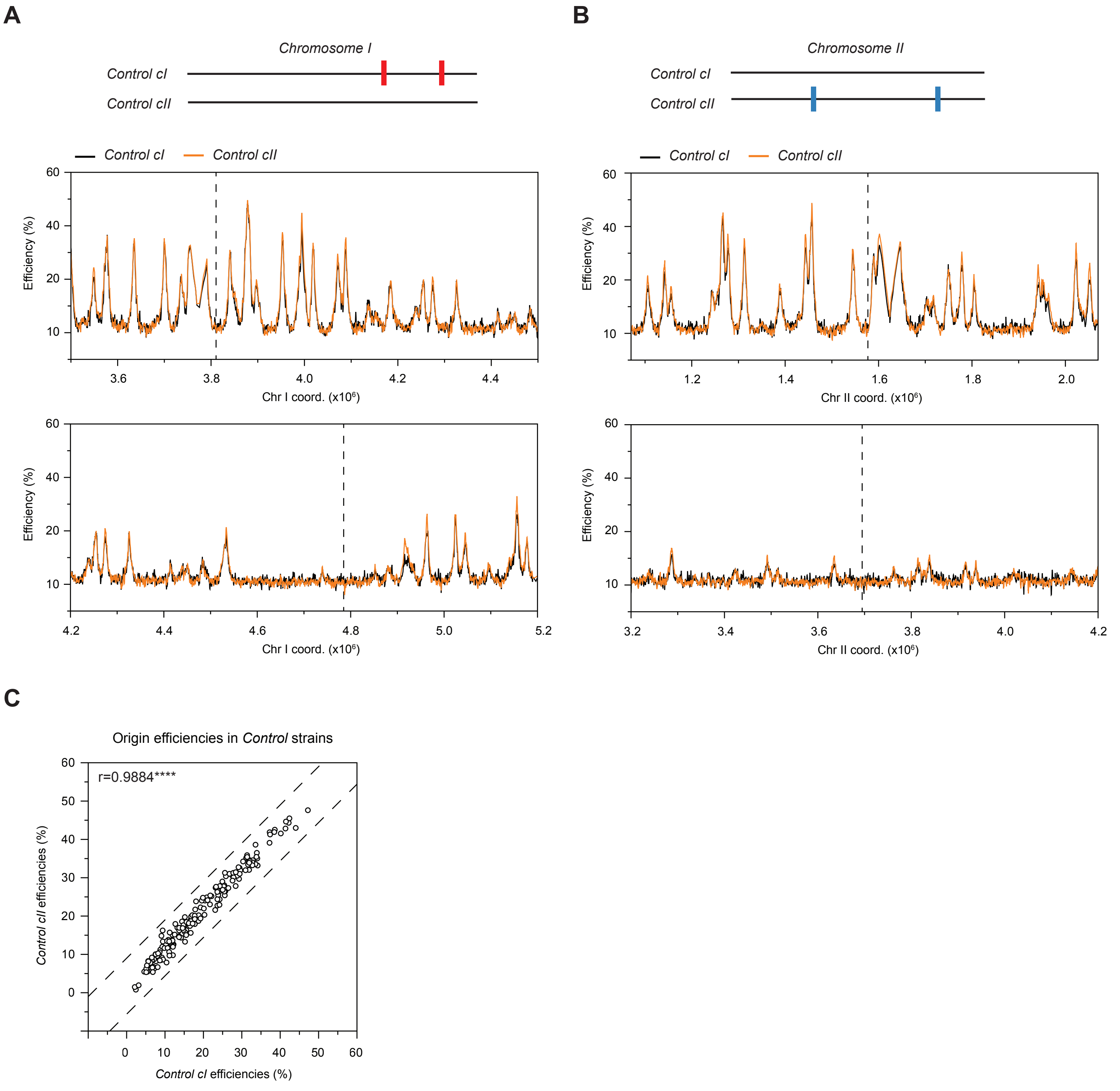

A
Chromosome I
Control cI
Control cII
Control cI
Control cII
Efficiency (%)
60
40
20
10
3.6
3.8
4.0
4.2
4.4
Chr I coord. (x10⁶)
4.2
4.4
4.6
4.8
5.0
5.2
B
Chromosome II
Control cI
Control cII
1.2
1.4
1.6
1.8
2.0
Chr II coord. (x10⁶)
3.2
3.4
3.6
3.8
4.0
4.2
C
Origin efficiencies in Control strains
r=0.9884****
Control cII efficiencies (%)
Control cI efficiencies (%)
0
10
20
30
40
50
60

Figure S4

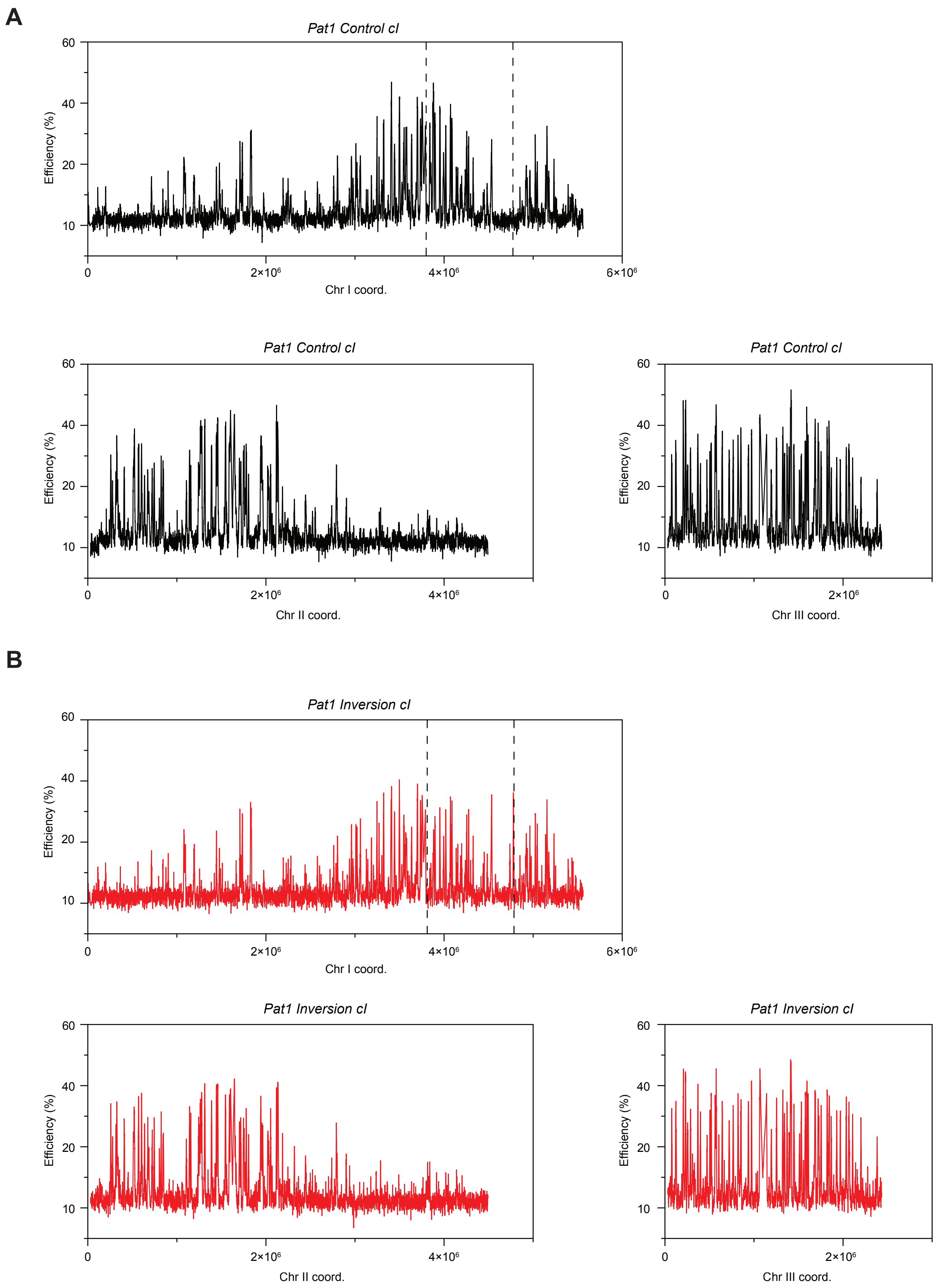
A
Pat1 Control cl
Efficiency (%)
60
40
20
10
0
2×10⁶
4×10⁶
6×10⁶
Chr I coord.
Pat1 Control cl
Chr II coord.
Pat1 Control cl
Chr III coord.
B
Pat1 Inversion cl
Chr I coord.
Pat1 Inversion cl
Chr II coord.
Pat1 Inversion cl
Chr III coord.

Figure S4

C

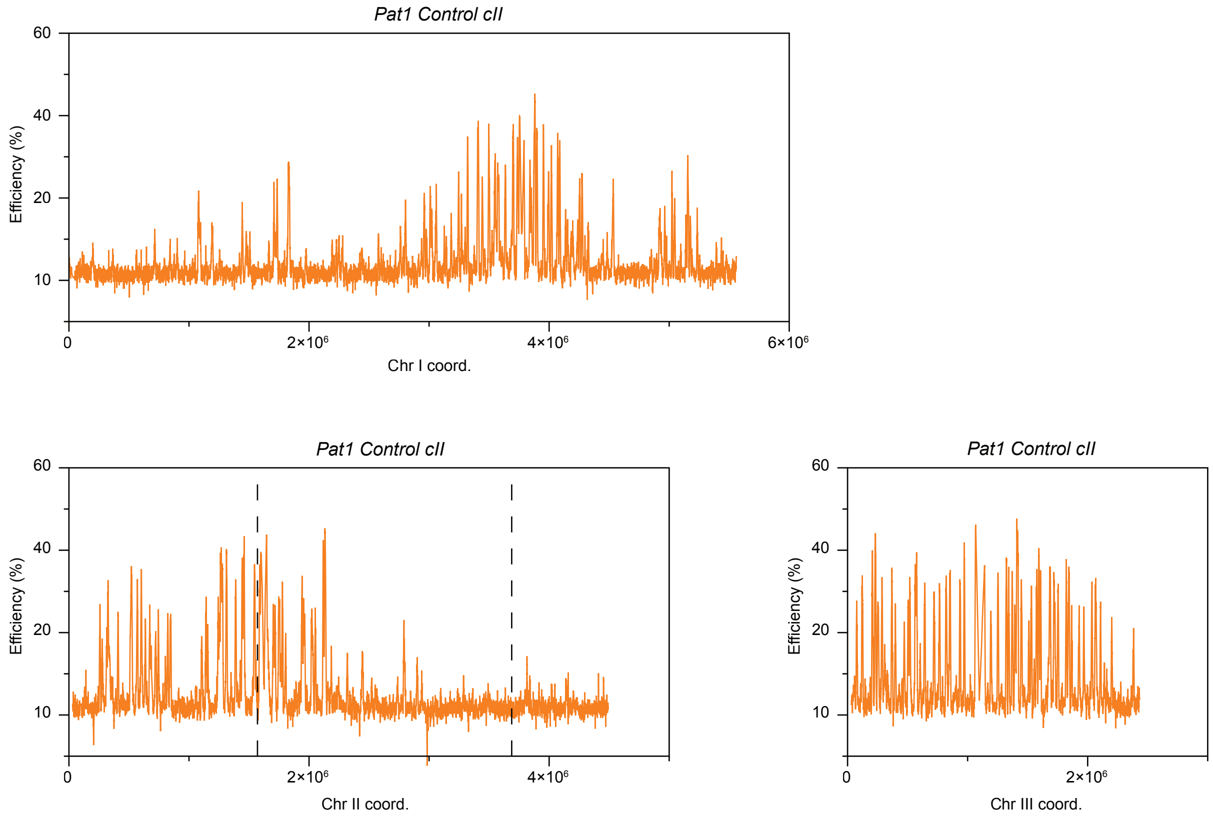
Pat1 Control cII
Efficiency (%)
60
40
20
10
0
2×10⁶
4×10⁶
6×10⁶
Chr I coord.
Pat1 Control cII
Chr II coord.
Pat1 Control cII
Chr III coord.

D

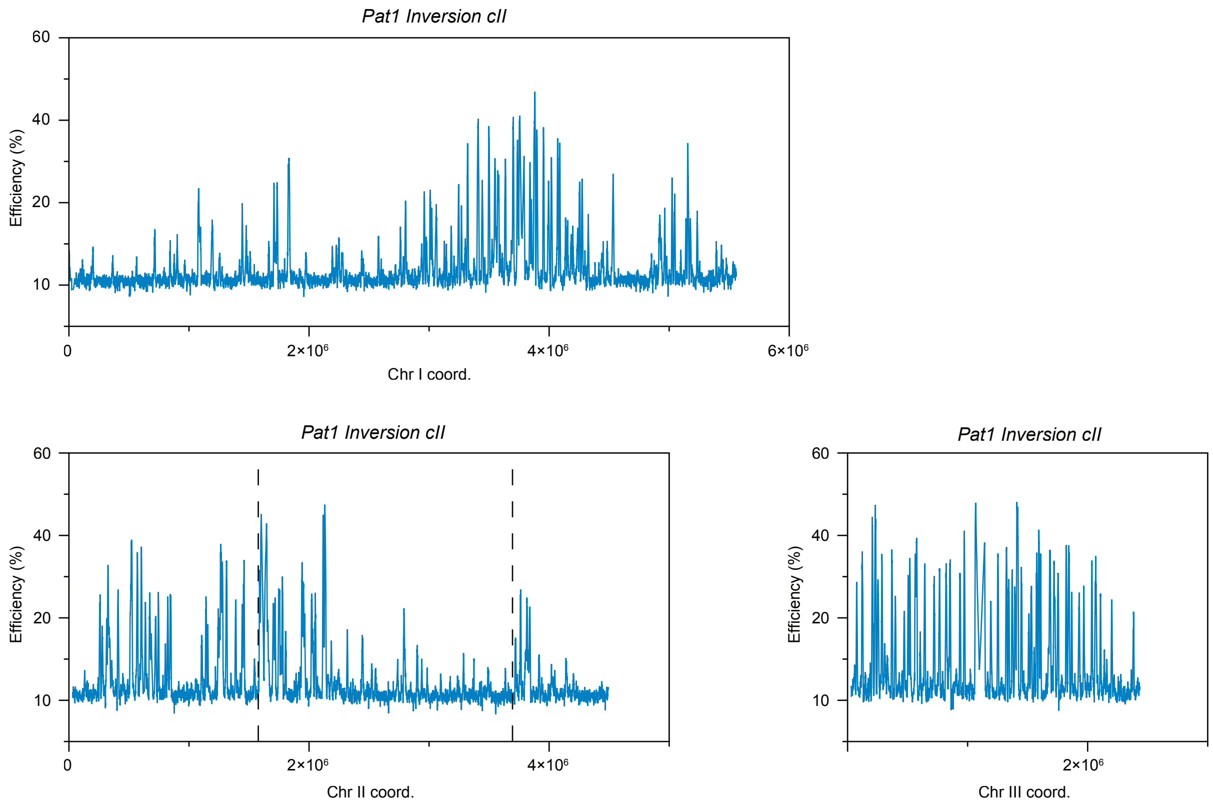
Pat1 Inversion cII
Efficiency (%)
60
40
20
10
0
2×10⁶
4×10⁶
6×10⁶
Chr I coord.
Pat1 Inversion cII
Chr II coord.
Pat1 Inversion cII
Chr III coord.

Figure S5

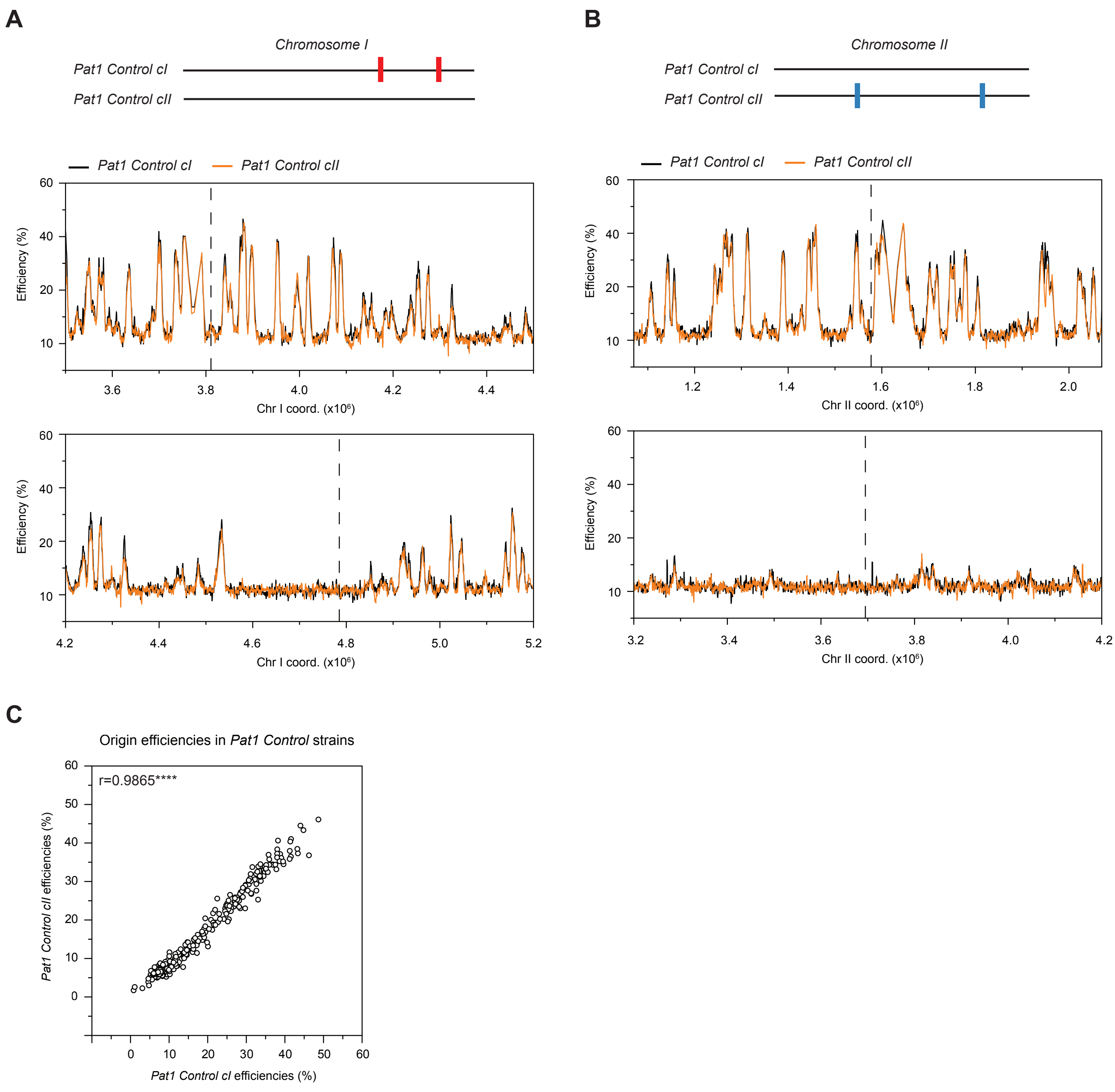
A
Chromosome I
Pat1 Control cI
Pat1 Control cII
Pat1 Control cI
Pat1 Control cII
Efficiency (%)
Chr I coord. (x10⁶)
B
Chromosome II
Pat1 Control cI
Pat1 Control cII
Efficiency (%)
Chr II coord. (x10⁶)
C
Origin efficiencies in Pat1 Control strains
r=0.9865****
Pat1 Control cII efficiencies (%)
Pat1 Control cI efficiencies (%)

Figure S6

A

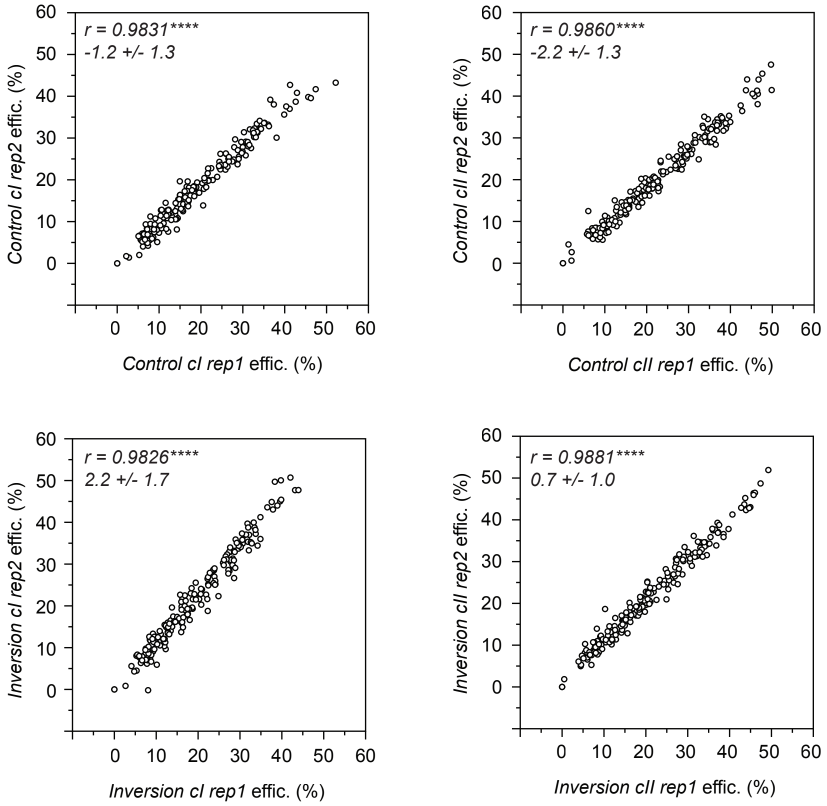
r = 0.9831****
-1.2 +/- 1.3
Control cI rep2 effic. (%)
Control cI rep1 effic. (%)
r = 0.9860****
-2.2 +/- 1.3
Control cII rep2 effic. (%)
Control cII rep1 effic. (%)
r = 0.9826****
2.2 +/- 1.7
Inversion cI rep2 effic. (%)
Inversion cI rep1 effic. (%)
r = 0.9881****
0.7 +/- 1.0
Inversion cII rep2 effic. (%)
Inversion cII rep1 effic. (%)

B

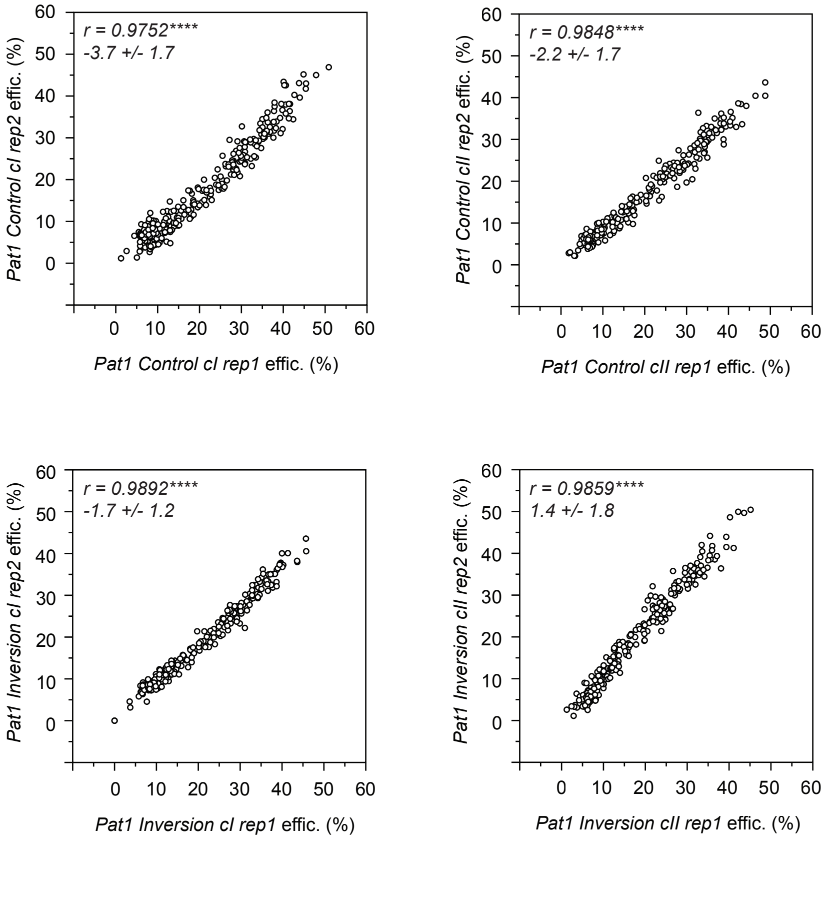
r = 0.9752****
-3.7 +/- 1.7
Pat1 Control cI rep2 effic. (%)
Pat1 Control cI rep1 effic. (%)
r = 0.9848****
-2.2 +/- 1.7
Pat1 Control cII rep2 effic. (%)
Pat1 Control cII rep1 effic. (%)
r = 0.9892****
-1.7 +/- 1.2
Pat1 Inversion cI rep2 effic. (%)
Pat1 Inversion cI rep1 effic. (%)
r = 0.9859****
1.4 +/- 1.8
Pat1 Inversion cII rep2 effic. (%)
Pat1 Inversion cII rep1 effic. (%)

Figure S7

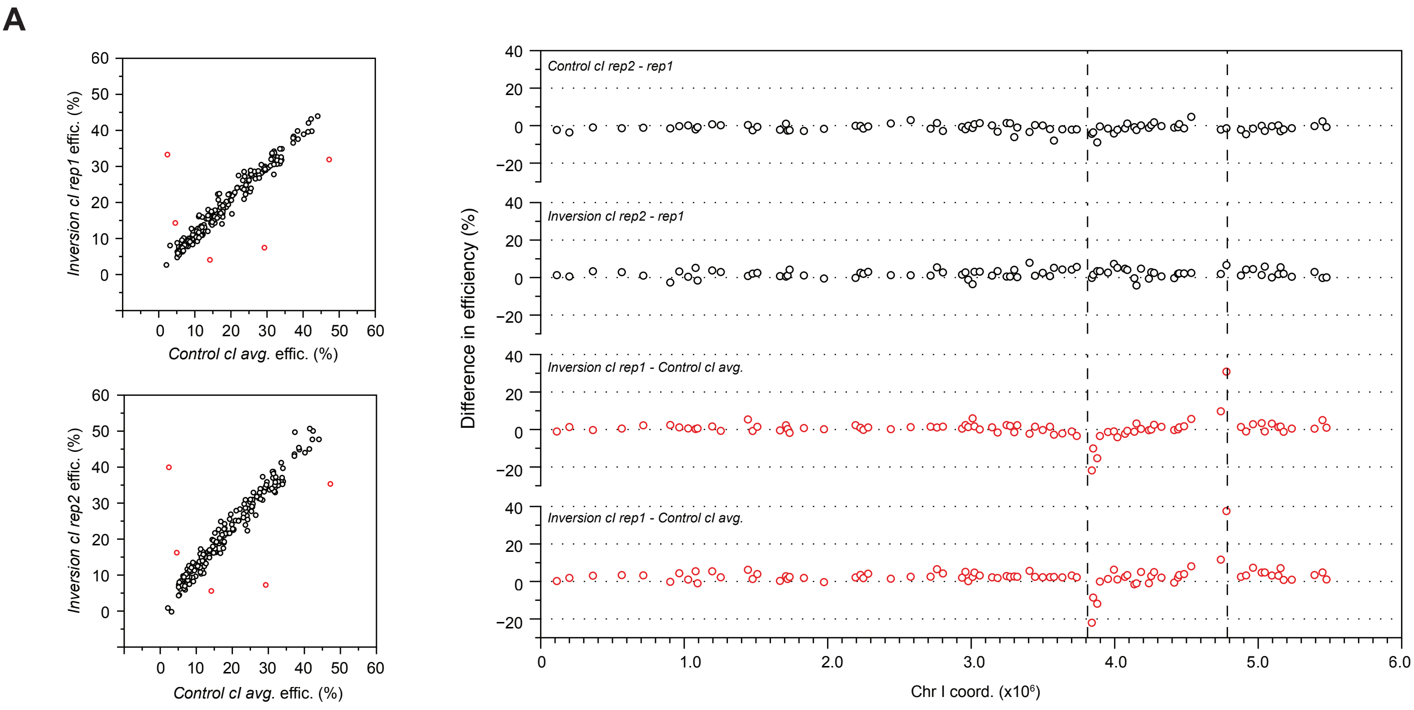
A
Inversion cI rep1 effic. (%)
Control cI avg. effic. (%)
Inversion cI rep2 effic. (%)
Control cI avg. effic. (%)
Control cI rep2 - rep1
Inversion cI rep2 - rep1
Inversion cI rep1 - Control cI avg.
Inversion cI rep1 - Control cI avg.
Difference in efficiency (%)
Chr I coord. (x10⁶)

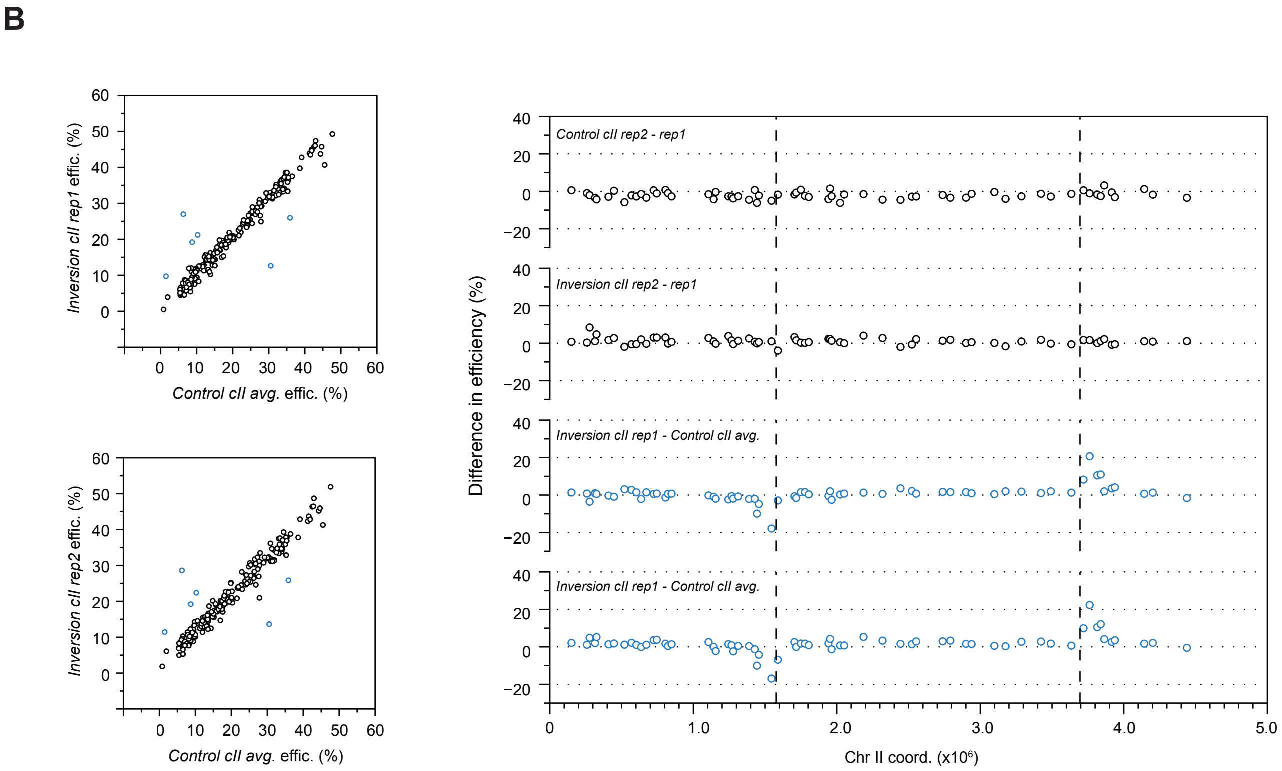
B
Inversion cII rep1 effic. (%)
Control cII avg. effic. (%)
Inversion cII rep2 effic. (%)
Control cII avg. effic. (%)
Control cII rep2 - rep1
Inversion cII rep2 - rep1
Inversion cII rep1 - Control cII avg.
Inversion cII rep1 - Control cII avg.
Difference in efficiency (%)
Chr II coord. (x10⁶)

Figure S7

C

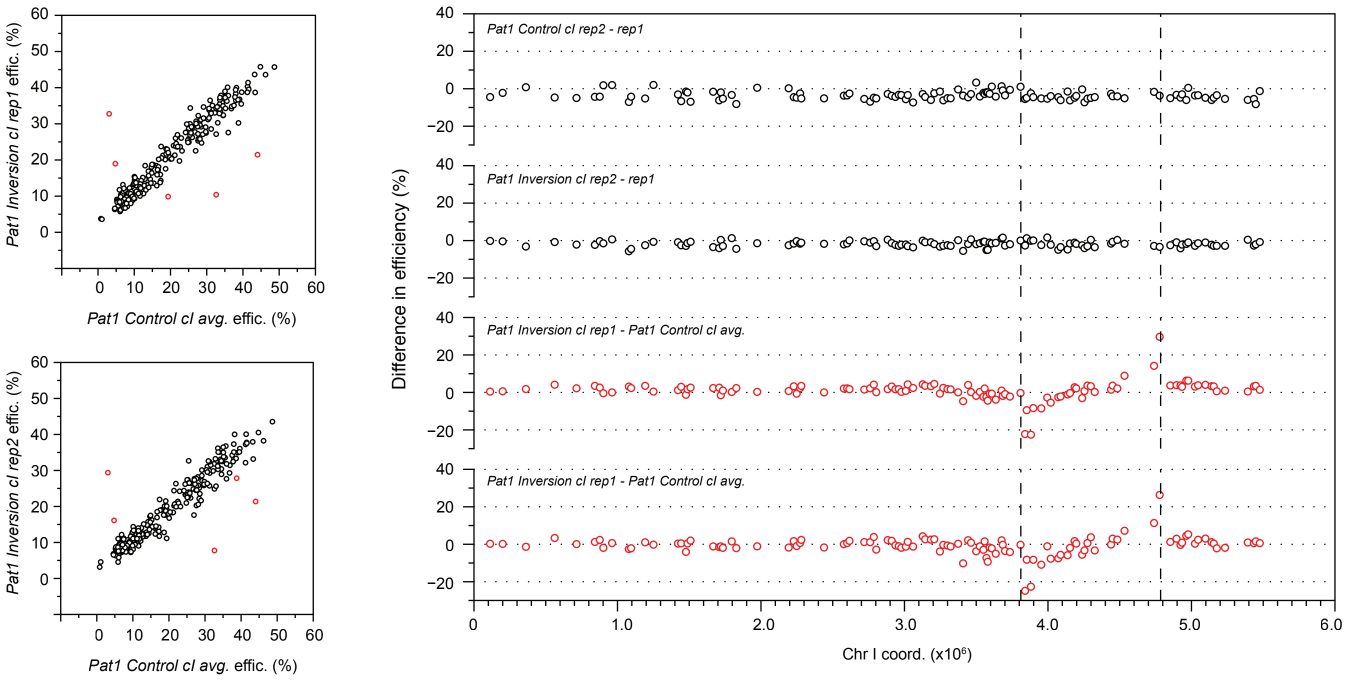
Pat1 Inversion cI rep1 effic. (%)
Pat1 Control cI avg. effic. (%)
Pat1 Inversion cI rep2 effic. (%)
Pat1 Control cI avg. effic. (%)
Difference in efficiency (%)
Pat1 Control cI rep2 - rep1
Pat1 Inversion cI rep2 - rep1
Pat1 Inversion cI rep1 - Pat1 Control cI avg.
Pat1 Inversion cI rep1 - Pat1 Control cI avg.
Chr I coord. (x10⁶)

D

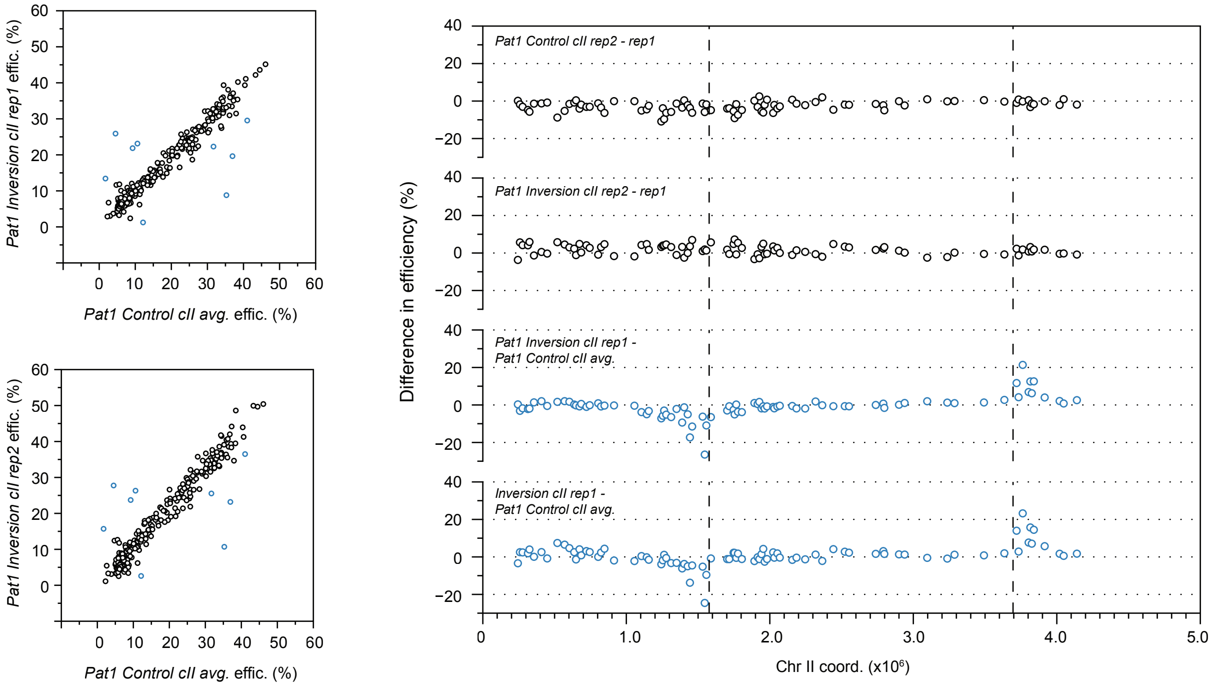
Pat1 Inversion cII rep1 effic. (%)
Pat1 Control cII avg. effic. (%)
Pat1 Inversion cII rep2 effic. (%)
Pat1 Control cII avg. effic. (%)
Difference in efficiency (%)
Pat1 Control cII rep2 - rep1
Pat1 Inversion cII rep2 - rep1
Pat1 Inversion cII rep1 -
Pat1 Control cII avg.
Inversion cII rep1 -
Pat1 Control cII avg.
Chr II coord. (x10⁶)

Supplementary Figure legends

Figure S1. Strategy for generating chromosomal rearrangements. A) Replication profile of a wild-type haploid strain in vegetative growth (Top) or exiting quiescence (Middle). Data are from Fig. 1C and 1D in Wu and Nurse, 2014. x-axis: chromosome coordinates, y-axis: origin efficiencies. Dashed lines represent the locations of the *loxP* insertions (ChrI: 3810766 and 4785016; ChrII: 1577150 and 3694900). Bottom: Schematic of the engineered inversions. Origin efficiencies are indicated by the gradient; darker colors correspond to higher efficiencies. The objective was to alter the chromosome context by disrupting and juxtaposing distinct efficiency domains. The diagram shows how the replication program would appear after the simple inversion of a genomic fragment, in the case that it has no effects on DNA replication. Triangles indicate the positions of centromeres. B) Experimental design for generating chromosomal inversions. Two cassettes were constructed: one contains a *loxP* site flanked by the TEF promoter and the *LEU2* gene from *S. cerevisiae*; a second contains *loxP*, the *ura4* gene from *S. pombe*, and the kanMX ORF. These cassettes were amplified and inserted in distinct loci by homologous recombination. Transient expression of Cre recombinase promotes recombination between the *loxP* sites. Strains with productive rearrangements place the TEF promoter 5' to the kanMX ORF, resulting in resistance to G418. C) Sites of *loxP* insertion in the indicated positions on chromosomes I and II. Images are taken from PomBase [1].

Figure S2. Replication profiles of the first post-quiescence S phase in different genetic backgrounds. A-D) Genome-wide origin efficiencies of *Control cI* (*A*, black), *Inversion cI* (*B*, red), *Control cII* (*C*, orange), and *Inversion cII* (*D*, blue). To allow direct comparison with the *Control* strains, the data for *Inversion cI* and *Inversion cII* are plotted using the original, pre-rearrangement coordinates. Dashed lines: inversion endpoints. x-axis: chromosome coordinates, y-axis: origin efficiencies.

Figure S3. Origin usage in the *Control* strains. A-B) Top: Schematic of the *loxP* sites present in the indicated chromosomes in the *Control* strains (red boxes: sites in *Control cI*; blue boxes: sites in *Control cII*). Bottom panels: Detailed view of the replication profiles surrounding the inversion endpoints (dashed lines) in *Control cI* (black) and *Control cII* (orange). x-axis: chromosome coordinates, y-axis: origin efficiencies. C) Comparison of origin efficiencies in *Control cII* (y-axis) vs. *Control cI* (x-axis). Each point represents an origin. The Pearson's correlation coefficient (r) indicates a strong correlation between efficiencies in the two backgrounds. ****: p-value < 0.0001.

Figure S4. Replication profiles of pre-meiotic S phase in the different genetic backgrounds. Genome-wide origin efficiencies of *Pat1 Control cI* (*A*, black), *Pat1 Inversion cI* (*B*, red), *Pat1 Control cII* (*C*, orange), and *Pat1 Inversion cII* (*D*, blue). To allow direct comparison with the *Pat1 Control* strains, the data for *Pat1 Inversion cI* and *cII* are plotted using the original, pre-rearrangement coordinates. Dashed lines: inversion endpoints. x-axis: chromosome coordinates, y-axis: origin efficiencies.

Figure S5. Replication profiles of the *Pat1 Control* strains. A-B) Top: Schematic of the *loxP* sites present in the indicated chromosomes in the *Pat1 Control* strains (red boxes: sites in *Pat1 Control cI*; blue boxes: sites in *Pat1 Control cII*). Bottom panels: Detailed view of the replication profiles surrounding the inversion endpoints (dashed lines) in *Pat1 Control cI* (black) and *Pat1 Control cII* (orange). x-axis: chromosome coordinates, y-axis: origin efficiencies. C) Comparison of origin efficiencies in *Pat1 Control cII* (y-axis) vs. *Pat1 Control cI* (x-axis). Each point represents an origin. The Pearson's correlation coefficient (r) indicates a strong correlation between efficiencies in the two backgrounds. ****: p-value < 0.0001.

Figure S6. Comparisons of origin efficiencies from repeat experiments for each strain and condition. A-B) Graphs comparing the two biological repeats of origin mapping assays performed for each strain in a given condition in this study. The Pearson's correlation coefficient (r) for each comparison is displayed. ****: p < 0.00001. The median +/- median absolute deviation is also shown for each experiment. x- and y-axis: origin efficiencies of the indicated repeat (referred to as rep1 and rep2). These results show the high level of reproducibility of our datasets.

Figure S7. Reproducibility of the changes in origin efficiencies induced by chromosomal inversions. A-B) Post-quiesce S phase; C-D) Pre-meiotic S phase. Left panels: Comparisons of the origin efficiencies for individual repeats of a chromosomal inversion (y-axis) with the origin efficiencies determined from the average of the two repeats of the corresponding control strain (x-axis, data as in Figs. S3C and S5C). Each point represents an origin; those in red (*A, Inversion cI; C*, *Pat1 Inversion cI*) or in blue (*B, Inversion cII*; *D, Pat1 Inversion cII*) represent origins that were identified to show significant changes in Figures 3 and 6. Right panels: Right panels: Changes in replication initiation are reproducibly detected near the endpoints of chromosomal inversions. Only chromosomes harboring the inversions are shown. Dashed lines indicate rearrangement endpoints. To allow direct comparison with the *Control* strains, the *Inversion* strains are plotted using the original, pre-translocation coordinates. x-axis: chromosome coordinates; y-axis: origin efficiencies. Black circles: Differences in origin efficiencies between repeats of the indicated strains. Data from Figure S6 are plotted according to their chromosomal positions. Red and blue circles: Differences in origin efficiencies between individual repeats of the indicated inversion strain compared to the origin efficiencies determined from the average of the two repeats of the corresponding control strain. Data from the left panels in *A-D* are plotted according to their chromosomal positions.

References

1. Harris, M. A.; Rutherford, K. M.; Hayles, J.; Lock, A.; Bähler, J.; Oliver, S. G.; Mata, J.; Wood, V. Fission stories: using PomBase to understand Schizosaccharomyces pombe biology. *Genetics* **2022**, *220*.
